# Patients’ views on a subsidy card model for gluten-free food access: a qualitative study

**DOI:** 10.1186/s12913-025-13582-z

**Published:** 2025-10-29

**Authors:** Abubakar Sha’aban, Francesca Mazzaschi, Elizabeth Doe, Beti-Jane Ingram, Alison Jones, Emma Williams, Heather O’Sullivan, Andrew Evans, Adrian Edwards, Natalie Joseph-Williams

**Affiliations:** 1 Health and Care Research Wales Evidence Centre, Division of Population Medicine, School of Medicine, Cardiff University, Cardiff, UK; 2 Health and Care Research Wales Evidence Centre Public Partnership Group, Division of Population Medicine, School of Medicine, Cardiff University, Cardiff, UK; 3 Hywel Dda University Health Board, Carmarthen, Wales, UK; 4 Public Health Wales, Cardiff, UK; 5 Welsh Government, Cardiff, UK

**Keywords:** Subsidy card, Gluten-free, Prescriptions, Coeliac disease, Patient perspectives, Qualitative study

## Abstract

**Background:**

Coeliac disease requires strict adherence to a gluten-free diet to prevent health complications. While gluten-free foods (GFF) are traditionally available on the National Health Service (NHS) prescriptions, the cost and limited product range have prompted consideration of alternatives. Wales is exploring replacing prescriptions with a subsidy card scheme, piloted and implemented by the Hywel Dda University Health Board. This study explores potential service users’ views on the advantages and challenges of transitioning to a subsidy card scheme, as well as their priorities as individuals with coeliac disease.

**Methods:**

A qualitative study was conducted with 23 participants from six Welsh health boards where the subsidy card scheme has not been implemented. Participants included individuals eligible for GFF prescriptions and carers of eligible individuals. Recruitment was via social media and Coeliac UK’s Wales mailing list, with purposive sampling to ensure diversity. Data were collected through semi-structured interviews (April-July 2024) and analysed thematically using NVivo software.

**Results:**

Four themes and 20 subthemes were identified. Participants viewed the subsidy card as offering greater choice, autonomy in dietary management, and convenience compared to prescriptions, with the potential to better align with everyday shopping habits. Concerns centred on the card’s monetary value amid inflation, risks of technical or top-up failures, misuse, and limited retailer participation in rural areas. Sixteen participants expressed a clear interest in switching to the subsidy card system, six were ambivalent, and one expressed no interest. Participants also highlighted six key priorities that they felt were essential to improving GFF access and ensuring better support for people with coeliac disease. These priorities included: ease of access, variety and freedom of choice, a tasty and healthy diet, consistency, cost, and better awareness and education of coeliac disease.

**Conclusions:**

The subsidy card model presents a promising, cost-effective, and patient-centred alternative to prescriptions, offering greater dietary autonomy and alignment with consumer practices. However, its success will depend on ensuring equity across geographic areas, addressing infrastructure and retailer participation, safeguarding nutritional adequacy, and maintaining prescription access during transition. Careful implementation will be essential to support diverse patient needs and optimise the scheme’s success.

**Supplementary Information:**

The online version contains supplementary material available at 10.1186/s12913-025-13582-z.

## Background

Coeliac disease is a lifelong autoimmune condition that requires strict adherence to a gluten-free diet to prevent long-term health complications. To support access, gluten-free foods (GFF) have been available on prescription in the UK since the late 1960s, a measure introduced to support dietary adherence when such foods were scarce in shops [1]. However, with the increased availability of GFF in supermarkets, alternative models of GFF supply are being considered by some health service providers. These include NHS payors subsidising the purchase of GFF through models which have additional benefits for patients and the NHS, over GFF prescribing by general practitioners. For subsidy models to be successful, it is important to understand patients’ perceptions on benefits, disadvantages, and barriers to implementation.

A subsidy card scheme involves an NHS payor providing patients with a pre-payment card which is ‘topped up’ by the NHS at regular intervals [2]. Cards can be used at retailers to purchase GFF or other foods. In general, the price paid by the NHS for prescribed GFF is higher than the retail price of equivalent products. To contain costs, NHS payors have acted to restrict prescribed product choice. Although the prescription model is available to all eligible patients, some do not use it due to perceived restrictiveness (e.g., limited product choice) or the administrative burden of obtaining prescriptions. The increased availability and choice of GFF in supermarkets and other retailers, and higher costs to the NHS, have led to an interest in whether supporting patients to buy GFF directly, either complementing or replacing more limited prescribing of GFF [3], could improve patient experience, and reduce NHS costs [4].

In Wales, healthcare is structured through seven Local Health Boards which are statutory NHS bodies responsible for planning, commissioning, and delivering services (such as dental, pharmacy, and mental health) for their populations [5, 6]. In 2018, a Welsh health board introduced an opt-in pilot in nine General Practices. Participants received pre-paid Mastercards, topped up every three months according to national prescribing guidelines for nutritional requirements, which were calculated by age and gender [7]. Top-up amounts varied by demographic group: children received £24–63 per 3 months; adult women £42 per 3 months; and adult men £54 per 3 months (unpublished scheme data). After full implementation in 2019, there are plans to extend the scheme to all other health boards in Wales [8]. Prior to extending the scheme, it was considered critical to understand the views of people with coeliac disease who were not involved in the pilot scheme. This study aimed to explore patients’ views on using a pre-paid subsidy card in place of GFF prescriptions. Specifically, we identified patient-perceived benefits and disadvantages of subsidy card schemes for accessing GFF and potential barriers to implementation, which will be used to inform the scheme’s future roll-out plans.

## Methods

### Study design

This qualitative study used semi-structured interviews to explore the views of patients with coeliac disease who would be eligible for the subsidy card scheme in a potential future roll-out across health boards in Wales. Recruitment processes and documents were developed with our Public Partner and stakeholders to ensure they were feasible, understandable, and relevant. We have prepared this report to adhere to the Consolidated criteria for Reporting Qualitative research (COREQ) checklist (see Supplementary Material 1).

Inclusion criteria were: (i) individuals diagnosed with coeliac disease and receiving or eligible to receive prescriptions for gluten-free products under any Welsh health board (and who would thus be eligible for a future roll-out of the scheme); and (ii) individuals responsible for acquiring gluten-free products for someone with a coeliac disease diagnosis under any Welsh health board. Exclusion criteria were: (i) those residing outside of Wales; (ii) individuals under the age of 18; and (iii) those following a gluten-free diet without a diagnosis of coeliac disease or dermatitis herpetiformis.

### Participant recruitment

Our sampling frame included Welsh residents (excluding patients in the health board where subsidy cards had been rolled out) who are eligible to receive GFF through NHS prescriptions or acquire GFF via prescription for someone else. Participants were recruited primarily through the social media ‘X’ (formerly known as Twitter) accounts of Health and Care Research Wales Evidence Centre, and The Wales Centre for Primary and Emergency Care Research (PRIME Centre Wales) and through relevant Facebook groups for people with coeliac disease (e.g., Coeliac Disease for Beginners). These platforms were chosen for their wide reach across Wales and their ability to connect with younger and middle-aged users who are active online. Recruitment information was also shared via Coeliac UK’s mailing list with its Wales-based members. Coeliac UK is a charity supporting individuals who need to live gluten-free. This multi-channel approach maximised recruitment efficiency and access to a broad pool of potential participants.

Interested individuals were directed to an online screening and demographic survey, which collected information on gender, age group, health board area, eligibility for NHS GFF in Wales, GFF access method, disability status, highest educational level, employment status, personal income range (previous year), and ethnicity, and three questions assessing digital literacy [9]. A total of approximately 70 eligible individuals completed the screening and demographic survey. From this pool, 40 participants were purposively selected to achieve diversity and maximum variation [10] ensuring diversity across the demographic and socioeconomic variables collected in the screening survey. These individuals were subsequently contacted to schedule an interview at a mutually convenient time. In advance of the interview, participants were provided with consent forms for review, signature, and return. Of the 40 individuals invited, 23 consented and participated in the interviews.

### Data collection

Semi-structured interviews were conducted online via Zoom or Microsoft Teams between April and July 2024. Two trained qualitative researchers (AS – male, PhD; FM – female, PhD) conducted the interviews. Both were Research Associates and had formal training and prior experience in qualitative interviewing. They had no pre-existing relationships with participants, and participants were informed that the interviewers were researchers studying healthcare access and patient perspectives. The interviews lasted approximately 30–45 min and were audio-recorded with participant consent. Only the participant and interviewer were present at each interview. The researchers used an interview guide (see Supplementary Material 2) to guide the discussion and ensure key topics were explored. The interview schedule was developed with stakeholders, including our Public Partner (B-JI), to ensure the questions were accurate, accessible, and covered areas that mattered to people with coeliac disease. No repeat interviews were conducted, and transcripts were not returned to participants for checking. Field notes were written after each interview to capture context and any peculiar situations. Interviews continued until data saturation [11] was reached, i.e., until no new significant themes emerged. Saturation was achieved within the 23 participants who consented and took part in the study.

### Data analysis

Interviews were transcribed verbatim and transcripts imported into NVivo 12 pro Qualitative Analysis Software [12] for analysis. Thematic analysis [13] was applied to the data [14] by AS and FM; this is a systematic method used to identify patterns and themes within data. It involves a series of structured steps including familiarisation with the data, coding, developing a thematic framework, charting the data into the framework (including verbatim quotes), and interpreting the key themes.

The initial thematic coding framework (emerging themes from preliminary exploration of the data) was applied to a set of transcripts (*n* = 6) by FM and cross-checked by AS. The initial coding framework generated seven overarching themes. Several sub-themes were subsequently merged where overlap was identified, and additional inductive sub-themes were added during iterative review. The emerging frameworks were discussed with NJW and refined before applying the frameworks to the remainder of the transcripts. Themes were derived inductively. No participant checking of findings was conducted.

## Results

### Participants

We recruited 23 participants across the six health boards in Wales where the subsidy card scheme has not yet been rolled out. These individuals were drawn from the 40 invited following purposive sampling from the initial 70 who completed the screening and demographic survey. Overall, 87% (*n* = 20) of participants identified as female; 13% identified as male (*n* = 3). Participants reported a range of disability status, educational attainment, employment status, and personal income range (see Table 1**).** All 23 participants are eligible for NHS prescription of GFF in Wales, either for themselves (*n* = 17, 74%) or for others they care for (*n* = 6, 26%). Of the 23 participants, four chose not to receive prescriptions for GFF; we refer to these individuals as those who ‘do not use prescriptions’ in this paper. Participants age groups ranged from 18–24 to 75–84, with the most frequent age categories being 35–44 and 55–64 (Table 1).

**Table 1:** Demographic characteristics of study participants (*n* = 23)

Characteristic	Number of participants *n* (%)
**Age Range (Years)**	
18–24	2(9)
25–34	4(17)
35–44	5(22)
45–54	3(13)
55–64	5(22)
65–74	3(13)
75–84	1(4)
**Gender**	
Male	4(17)
Female	19(83)
**Personal Eligibility for NHS GFF Provision in Wales**	
Self-Eligible	16(70)
Responsible for an Eligible Child	6(26)
Responsible for an Eligible Spouse	1(4)
**Prescription use**	
Yes	19(83)
No	4(17)
**Disability Status**	
Mobility Impairment	1(4)
Chronic Illness	1(4)
Mental Health Condition	1(4)
None	20(88)
**Highest Educational Level Attained**	
School or College Lever Qualification	4(17)
University Level Qualification	19(83)
**Employment Status**	
Employed Full-time	15(65)
Self Employed	2(9)
Part-time Self Employed	1(4)
Homemaker	1(4)
Retired	4(17)
**Personal Income Last Year**	
£1 - £9,999	1(4)
£10,000-£24,999	8(35)
£25,000-£49,999	10(43)
£50,000-£74,999	3(13)
**Ethnicity**	
White	21(91)
White and Black Caribbean	2(9)

### Themes

Four overarching themes and 20 subthemes were identified: (1) Perceived advantages of the subsidy card (2), Potential concerns about the subsidy card (3), Views on switching to a subsidy card, and (4) Main priorities of people with coeliac disease.

- **Perceived advantages** included increased choice, greater autonomy in managing the condition, perceived improvements in diet quality, convenience and time-saving benefits, and more holistic support.
- **Concerns** centred on the monetary value of the card in the current economic climate, risk of increased taxpayer burden, potential misuse, technical or top-up failures, and challenges in rural areas where shops may be less familiar with the scheme.
- **Views on switching** reflected three positions: clear interest in adopting the subsidy card system, ambivalence about switching, and a small minority expressing no interest in switching.
- **Main priorities of people with coeliac disease** were ease of access, variety and freedom of choice, a tasty and healthy diet, consistency in availability, affordability, and better awareness and education about coeliac disease.

Quotations are presented in the results to illustrate themes and subthemes, capturing the perspectives of both prescription users and non-users.

### Perceived advantages of subsidy card

Participants identified several perceived advantages, highlighting its potential to improve various aspects of their experience of accessing GFF.

**Increased choice** was perceived as a significant benefit for many. Participants valued the potential to choose from a wide range of gluten-free products without the restrictions they currently experience with the prescriptions:

*So my hope would be that I would be able to choose the products that I want. And that there wouldn’t be a limit on you can only use it for bread or cereal or biscuits or whatever*,* that actually*,* there’s a range…you can use this against any gluten-free products in any shop. (P26_Do not use prescription)*

One participant who already complements their prescription items with items bought at the supermarket noted that it would afford them greater choice. Instead of choosing the cheaper option at the supermarket, which is what they do when they are self-paying, they could **choose what they prefer to eat**:

*I think a pre-paid card would mean more variety again*,* because a lot of times I’ll go in and say if I’m buying like a loaf*,* I will choose what is cheaper*,* rather than which one I prefer. I think it’d give me a lot more options. (P05_Prescription User)*

Others felt that the increased choice available to them would **improve their diet**.

*I’ll be having more of the product that I enjoy and ultimately eating better than what I am at the moment. (P03_Prescription User)*

The subsidy card is also perceived to give patients **greater autonomy in managing their condition.**

*I think a pre-payment card that didn’t depend on it being prescribed in a pharmacy getting it in*,* so you had more control over what you got when. (P23_Do not use prescription)*

The **convenience and time-saving benefits** of a subsidy card were also emphasised. Participants noted that being able to purchase gluten-free products directly from local stores, rather than dealing with the complexities of prescriptions, would be significantly more efficient:

*I can pick up the Warburton’s gluten-free bread in my local Tesco’s*,* I can walk five minutes down the road and pick it up. But to try and get it on prescription has taken me two months and numerous trips into town. So*,* it just*,* it doesn’t work. (P02_Caring for Child_Prescription User)*

Some felt that the subsidy card would also provide a **more holistic support system** that recognised their individual needs.

*Yeah. I mean I have to say that if we went down this route [of subsidy card]*,* I would feel much more supported in terms of the holistic needs of a person with coeliac disease. (P16_Caring for Child_Prescription User).*

### Concerns about the subsidy card

In addition to the perceived advantages, some participants noted potential concerns. One concern was the **value of the card within the broader economic climate.** Participants worried that the card might be impacted by inflation and price fluctuations, which could affect its value over time:

*I suppose the disadvantage would be that it would be subjective to inflation and to the price changes and therefore may go further for some individuals than it does in others. (P16_Caring for Child_Prescription User)*

Another perceived issue was the **increased taxpayer burden.** Participants expressed concern that a subsidy card system might lead to higher costs for taxpayers, as more people with coeliac disease might use the card compared to the current prescription system:

*If I’m being honest*,* you know*,* hand on heart*,* the disadvantage comes to the taxpayer. Because more people who are coeliac are likely to use a card*,* whereas fewer of us use the prescription offer. So I think the disbenefit is to the taxpayer. (P26_Do not use prescription)*

**Potential misuse** of the card was another concern. Participants worried about what they perceived as improper use (e.g. purchasing non-GFF items, although the scheme does allow for such alternative expenditure with the subsidy care) or fraud, although they acknowledged that there could be mechanisms to mitigate these issues.

*The disadvantage*,* I suppose*,* is if it wasn’t being used properly. But I mean there’s*,* there’s ways of controlling that*,* isn’t there? It can only be used to purchase certain items… (P02_Caring for Child_Prescription User)*.

**Potential top-up failures** were also noted as a possible disadvantage. Participants feared that technical issues or failures in adding funds to the card could leave them without the necessary products:

*Well*,* only if the top-up failed or something*,* if you were left there without anything on it when there should be stuff on it really. I mean*,* that would be a disadvantage. (P13_Prescription User)*

Additional barriers were noted for those who live in **rural locations.** Participants valued the convenience of prescriptions being available without needing to travel far, which might not be as feasible with a subsidy card.

*On the flip side*,* I think it is good with the prescriptions that it’s just there*,* and I have to go and get it. It can be a bit of a faff trying to access it [the process]*,* but at the end of the day*,* there is food for me that I don’t have to worry too much about having to travel too far for. So*,* living rurally [the prescription] is quite good. (P11_Prescription User)*

Finally, there were some concerns about **shops’ familiarity with the card.** Participants expressed concerns that some stores might not accept the subsidy card or might not be familiar with the system, which could limit where they could use it.

*Disadvantages would be if certain places didn’t accept it and that you were still restricted to certain locations to buy*,* you know*,* those products*,* which I think would still be fine. (P07_Prescription User)*

### Views on switching to a subsidy card

Participants had a range of views on switching to the subsidy card system. Sixteen participants expressed clear interest in switching to the subsidy card system, six were ambivalent, and only one explicitly mentioned a lack of interest in switching.

Some participants **wanted to switch to the new scheme**, should it become available, noting that they would prefer it over the prescription model:

*I mean*,* obviously*,* the card system…that sounds ideal. When I read about it*,* I was like*,* this seems like a really good idea*,* and I’d much prefer that. So if I were to go into the shop and choose what I wanted and then you’d pay for it with a sort of discount on the product. (P07_Prescription User)*

*The card*,* I think*,* is a fantastic idea…the dietician actually mentioned that*,* and she said*,* ‘There’s other Health Boards doing that.’ I was like*,* ‘Oh*,* that would work so much better.’ The current system just doesn’t work for anybody. It really doesn’t. (P02_Caring for Child_Prescription User)*

Others felt like it was a good idea, but they **required more information** about how the card is used before fully committing:

*It sounds a good idea*,* but how—how much is on that card? How…? What would be the discount? And Is it loaded with so much money*,* and then it gets knocked off each time you use it*,* or…? Probably*,* yes*,* if I got more information*,* you know*,* written down and just read it through. But it sounds like a good idea. (P08_Prescription User)*

*Well*,* I’d look at*,* you know*,* what terms*,* conditions and things. But yes*,* possibly. (P21_Prescription User)*

Some wanted **specific reassurance** that they would not be at a **financial disadvantage**:

*Depends how much it’s loaded with*. *(P22_Prescription User)*

*To have it*,* probably to have it inflation-linked every… you know*,* obviously not every three months*,* but at least every year or two years or something*,* so it could be assessed*,* and*,* if the prices keep rising as they are*,* it would end up in 10 years’ time being worthless if it didn’t change*,* you know. So it would have to be sort of reassessed every so often. I don’t know what sort of timescale*,* really*,* but I think if things like that were sorted out*,* it could work. (P13_Prescription User)*

**Opposition to the subsidy card** was expressed by one participant who cares for someone who is a prescription user. They preferred the current prescription system or had reservations about potential changes:

*Let me be honest*,* I won’t really accept it. I won’t. (P10_Caring for Spouse_Prescription User)*

### Main priorities of people with coeliac disease

In addition to their views on the subsidy card, participants also outlined six key priorities for improving access to GFF and better supporting people with coeliac disease.

One major priority for participants was **ease of access** to gluten-free products. Many participants emphasised the importance of obtaining gluten-free products easily and conveniently:

*Being able to access it…having the choice*,* you know*,* it’s products that he’ll eat and that we can get hold of them when we need to. (P02_Caring for Child_Prescription User)*

Another key concern was **variety and freedom of choice**, as participants wanted a broader selection of gluten-free options without having to compromise personal preferences:

*I think for me the main importance is to be able to access a good variety of food. Because… not just for children*,* adults like treats as well*,* I think that it’s still very limited. (P04_Caring for Child_Prescription User)*

*Being able to– just being able to buy the product I want and not having to make do with something else. (P24_Prescription User)*

Participants also stressed the importance of a **tasty and healthy diet**, wanting access to gluten-free products that are both nutritious and enjoyable to eat.

*Also*,* to have the choice of being able to have high quality*,* nice tasting*,* more normal foods because a lot of the times of the gluten-free things*,* they don’t often taste very nice*,* and there’s a very different texture. (P28_Prescription User)*

*I’d say actually a bit of a mixture of more healthy foods as well*,* cos I find a lot of the gluten-free stuff… it’s usually like biscuits. (P05_Prescription User)*

**Consistency** in availability was another crucial factor, as participants wanted reliable access to their preferred products.

*I think the most important thing to me is reliability. (P11_Prescription User)*

The **cost** of gluten-free products was also a significant issue, with many participants expressing the need for fairer pricing and consideration of financial impacts, especially for those eligible for free school meals.

*We’ve noticed a massive increase*,* not only that*,* the food cost of living and the cost of food going up… like normal spaghetti*,* 45p… Tesco’s gluten-free spaghetti 90p*,* like that’s double.” (P14 _Non-Prescription User)*.

*The paediatric specialist said to take her off school lunches completely [due to cross-contamination]. It’s not fair because she’s entitled to free school meals…. that has made it more difficult*,* and it’s more expense because we have to provide lunch every day. (P04_Caring for Child_Prescription User)*

Finally, participants highlighted the need for **better awareness and education about coeliac disease**, as increased understanding could improve their daily experiences.

*Suppose a wider education of—you know*,* that people understand. I mean*,* in church on a Sunday… I took my own gluten-free wafers*,* and I heard somebody scoff. And you think that’s not very nice. I’m sorry*,* do you want coeliac disease? (P21_Prescription User)*

*Absolutely*,* and we don’t eat out hardly ever because people don’t take it seriously. (P04_Caring for Child_Prescription User)*

## Discussion

This study provides valuable insights into patients’ perspectives on a subsidy card model for gluten-free food (GFF) access, identifying key advantages and concerns of a subsidy card scheme, views on switching to a subsidy card and main priorities of people with coeliac disease. While there is limited existing literature specifically exploring subsidy card schemes for GFF access, previous research has identified the significant financial and logistical burdens faced by individuals with coeliac disease in maintaining a strict gluten-free diet [15–17]. Our findings suggest that a well-designed subsidy card model could address several of these challenges and serve as a cost-effective, patient-centred solution for improving access to GFF.

One of the primary concerns raised by participants was ease of access to gluten-free products, a challenge that has been widely acknowledged in previous studies [18]. In Wales, where gluten-free prescriptions remain available, the roll out of a subsidy card scheme in all health boards presents a potential alternative that could simplify access for patients. Unlike prescription-based models that require GP involvement and may be restrictive in product range, the subsidy card allows individuals to obtain GFF directly from retailers, aligning more closely with consumer habits and reducing dependency on an overburdened healthcare system. This approach appears beneficial when compared to models such as those in England, where prescription support has been significantly reduced in many areas [17, 19].

The transition from prescriptions to subsidy cards offers potential cost-saving advantages for the NHS. By enabling patients to purchase GFF from retailers directly, this approach eliminates the need for prescription reimbursements, thereby reducing administrative expenses and dispensing fees traditionally associated with prescription handling. In 2017, NHS England spent £15.7 million on gluten-free food prescriptions, including costs for GP consultations and pharmacist dispensing fees [20]. Additionally, pharmacists could allocate their time to more critical medical tasks rather than managing food prescriptions, enhancing overall efficiency. Similar cost-reduction mechanisms have been noted in other healthcare reforms aimed at streamlining prescription-related processes​ [21].

The issue of variety and freedom of choice remains a key priority for individuals with coeliac disease, as highlighted in our study. Previous research has demonstrated that restricted product availability can contribute to dietary monotony and potential health implications among those on a gluten-free diet [16, 22]. Limited choice often leads individuals with celiac disease to rely heavily on packaged gluten-free products, such as snacks and biscuits, which are typically higher in fats, sugars, and salt compared to their gluten-containing counterparts [22, 23]. Frequent consumption of these nutrient-poor products may reduce overall dietary quality and increase the risk of nutrition-related chronic conditions, including obesity and cardiovascular disease [22, 24]. Recent evidence also shows that greater dietary variety is strongly associated with improved food-related quality of life for people with coeliac disease [25], strengthening our finding that variety is integral to patient wellbeing. A subsidy card model, by allowing individuals to select from a wider range of products available in supermarkets and pharmacies, offers greater dietary autonomy compared to prescription-based models, which are often limited in scope. This aspect differentiates it from direct food provision systems such as those in Spain, where individuals receive a predetermined selection of gluten-free staples, also potentially limiting dietary flexibility [26].

While a subsidy card offers increased variety and autonomy, it is important to recognise that prescription-based gluten-free foods in the UK have often been fortified with key micronutrients such as calcium and iron, contributing to improved dietary adequacy in people with coeliac disease. In contrast, many commercially available gluten-free products remain unfortified [27], which may increase the risk of micronutrient deficiencies if prescriptions are entirely withdrawn. This issue aligns with recent findings comparing the nutrient composition of prescription-only and retail gluten-free foods in England, which show marked differences in fortification [28]. Ensuring that subsidy card models do not compromise nutritional quality will therefore require either closer engagement with manufacturers to expand fortification of retail products or parallel nutritional guidance for patients when making food choices.

Consistency in the availability of GFF was also highlighted as a major concern by participants, echoing findings from previous studies that have reported fluctuations in product availability at different shops [15]. While the availability of gluten-free food in mainstream supermarkets has improved in recent years [17], access in budget supermarkets and corner shops remains poor, and gluten-free products continue to be significantly more expensive compared to standard alternatives [15, 29]. A subsidy card model, if implemented effectively, could help address these disparities by enabling individuals to purchase gluten-free products from a wider range of retailers.

Cost remains one of the most significant barriers to maintaining a gluten-free diet, as gluten-free products are consistently more expensive than their gluten-containing counterparts [29]. According to the Coeliac UK 2024 report [29], the cheapest gluten-free loaf was found to cost more than six times that of a gluten-containing equivalent, and a weekly gluten-free food shop can be up to 35% more expensive. The same report compared average prices of gluten-free and gluten-containing foods (gram for gram) from March 2022 to March 2024, and found the following: bread loaves were 4.5 times more expensive; plain flour 2.0 times; bread rolls 3.1 times; pasta 2.0 times; crackers 1.7 times; and cereals 2.1 times [29]. While prescriptions provide a financial safety net in some regions, they are not universally available, as demonstrated by policy variations across the UK [30]. In Scotland and Northern Ireland, prescriptions for gluten-free products remain accessible, whereas in England, availability varies by local Integrated Care Board policies [30]. Internationally, different approaches have been implemented, such as tax deductions in Canada [26] and direct food provision in Spain and New Zealand [19, 26]. Compared to these models, a subsidy card offers a more flexible and dignified approach by integrating gluten-free purchases into regular shopping routines while still providing financial support.

Finally, better awareness and education on coeliac disease remains an ongoing priority, as misinformation and lack of understanding continue to impact daily life for individuals adhering to a strict gluten-free diet. Previous research has shown that social stigma and a lack of awareness in food service establishments contribute to anxiety and isolation among people with coeliac disease [31]. While a subsidy card scheme primarily addresses financial and access-related concerns, complementary efforts in public awareness campaigns and professional training for food industry staff would be necessary to improve the overall experience for those living with coeliac disease.

### Implications for policy and practice

The subsidy card model presents a promising alternative to existing GFF access strategies. Most concerns identified in this study are addressable through enhanced communication, better infrastructure (e.g., a user-friendly app), and inclusion of more retailers, particularly those offering affordable or culturally-specific options. Such steps could help ensure the subsidy card scheme delivers equitable benefits across diverse user groups and settings. We present some possible solutions and next steps in attempting to address the concerns and challenges in Box 1.

**Box 1:** Potential solutions to perceived concerns and challenges

Perceived concerns/challenges	Potential solutions/next steps
**Economic concerns**: Potential concerns over the card’s value due to inflation and increased cost of living	**Card value reviews**: Regularly review the card’s value to address inflation and market fluctuations. **Communication campaign**: Communicate the review period/process to service users and highlight the broader benefits of scheme.
**Technical issues**: Problems with topping up the card or system failures.	**Reliable technical support**: Ensure reliable card top-up processes and offer clear guidance about how to access customer support for technical issues.
**Retailer acceptance**: Uncertainty on which retailers accept the card. Not all retailers, especially online or budget stores, accept the card, limiting product availability and lower cost alternatives.	**Expanded retailer network**: Regularly review the list of participating retailers, and consider the inclusion of lower-cost stores, whilst balancing issues of potential misuse. **Communication campaign**: Communicate the list of stores that accept the card, ideally localised so that it is relevant and informative to the service user. This could help service users to plan their shopping trips more effectively.
**Cultural dietary needs**: Difficulty finding culturally specific gluten-free products.	**Cultural product partnerships**: Partner with additional retailers to offer culturally specific gluten-free options.
**Potential misuse**: Risk of card misuse for non-gluten-free items or the card being stolen.	**Audit and review** - appropriate use of the card will be set out in the documentation used in signing up for the card. The use of the card may be audited by NHS Wales. Patients should report the loss or theft of the card to NHS Wales to ensure that the card is cancelled. How to report lost or stolen cards is already in place (2)
**Geographic limitations**: Rural areas may have fewer options and less benefit from the card compared to urban areas.	**Prescription continuity**: Continue using prescriptions in areas of need, which could include more rural areas or for those with limited mobility.

### Strengths and limitations

This research captures a diverse range of participant experiences, offering valuable insights into the varied challenges faced by both patients with coeliac disease and parents of patients with coeliac disease, including those in rural areas. The study involved participants with different experiences, including those on the prescription system and those eligible but choosing not to use prescriptions. The qualitative methods allowed for an in-depth exploration of participants’ needs and the underlying reasons for their preferences, providing a comprehensive view of the challenges and potential solutions in accessing GFF.

We also took several steps to enhance the trustworthiness of our analysis, including independent coding by two researchers, cross-checking, and stakeholder input during development of the interview guide. However, some limitations must be acknowledged. First, transcripts were not returned to participants for comment and findings were not participant-validated, which may limit respondent validation of themes. Second, the interviewer–participant dynamic may have influenced responses; although interviewers had no prior relationships with participants, their professional backgrounds as health researchers may have shaped the direction of probing questions. Third, the reliance on online recruitment may have excluded individuals with lower digital literacy or limited internet access, potentially biasing the sample toward more digitally engaged individuals.

Also, those less connected with Coeliac UK and related patient advocacy groups may have been inadvertently excluded. This bias may have led to the unintentional exclusion of older, less digitally engaged, ethnic minority individuals or those from more deprived areas. Therefore, the findings presented may not accurately showcase the full range of barriers to uptake seen in the population as a whole. Future work should also explore the views of those who do not typically self-select for research participation and also use offline methods for data collection. Further, given the importance of geographical location, it would be important to target people living in rural communities to explore their views. This will allow for a more nuanced understanding of the opinions and experiences of those requiring GFFs.

## Conclusions

This study highlights the potential of a subsidy card scheme to enhance the quality of life for individuals with coeliac disease by increasing dietary choice, convenience, and autonomy. However, the transition from a prescription-based system to subsidy cards poses challenges, including geographic disparities, economic feasibility, and the risk of unintended consequences, such as increased healthcare costs. Addressing these concerns through targeted measures, such as improved infrastructure, expanded retailer partnerships, and clear communication, is essential to ensure equitable and sustainable implementation. Future roll-out plans must consider diverse patient needs and ensure that both systems remain complementary to provide holistic support for coeliac care across Wales. Future research should explore long-term patient satisfaction with the subsidy card model and its comparative effectiveness against other international approaches to GFF support. Given the limited published research on subsidy card models for gluten-free food access, this study provides an important contribution to an emerging evidence base and will support further policy and service design discussions.

## Supplementary Information

Below is the link to the electronic supplementary material.

Supplementary Material 1

Supplementary Material 2

## Data Availability

De-identified data supporting this study’s findings may be made available, upon reasonable request to the corresponding author.

## Declarations

### Transparency declaration

The authors affirm that this manuscript is an honest, accurate, and transparent account of the study being reported.

## References

[1] Kurien M, Trott N, Sleet S, Sanders DS. Prescribing gluten-free foods in general practice. Br J Gen Pract. 2018;68(673):364–5. 30049756 10.3399/bjgp18X698045 PMC6058628

[2] NHS Wales Shared Services Partnership. How the card works 2019. Available from: https://nwssp.nhs.wales/ourservices/primary-care-services/our-services/gluten-free-foods-subsidy-card-service/how-the-card-works/

[3] AWMSG. All Wales Guide to Prescribing Gluten-free Products 2018. Available from: https://awttc.nhs.wales/files/guidelines-and-pils/all-wales-guide-to-prescribing-gluten-free-products-pdf/

[4] Department of Health & Social Care. Consultation on the National Health Service (General Medical Services Contracts) (Prescription of Drugs etc.) (Amendment) Regulations 2018- Gluten Free Food on NHS Prescription in England. 2018.

[5] NHS Confederation. How is the NHS structured in Wales? Welsh NHS Confederation. 2024. Available from: https://www.nhsconfed.org/articles/how-nhs-structured-wales

[6] Welsh Government. NHS Wales health boards and trusts 2020 updated 3 February 2023. Available from: https://www.gov.wales/nhs-wales-health-boards-and-trusts?

[7] Coeliac UK. The National Prescribing Guidelines nd. Available from: https://www.coeliac.org.uk/information-and-support/coeliac-disease/once-diagnosed/prescriptions/how-much-should-be-prescribed/

[8] NHS Wales Shared Services Partnership. Gluten free foods subsidy card service 2019. Available from: https://nwssp.nhs.wales/ourservices/primary-care-services/our-services/gluten-free-foods-subsidy-card-service/

[9] Nelson LA, Pennings JS, Sommer EC, Popescu F, Barkin SL. A 3-Item measure of digital health care literacy: development and validation study. JMIR Form Res. 2022;6(4):e36043. 35486413 10.2196/36043 PMC9107049

[10] Vasileiou K, Barnett J, Thorpe S, Young T. Characterising and justifying sample size sufficiency in interview-based studies: systematic analysis of qualitative health research over a 15-year period. BMC Med Res Methodol. 2018;18(1):148. 30463515 10.1186/s12874-018-0594-7 PMC6249736

[11] Hennink M, Kaiser BN. Sample sizes for saturation in qualitative research: A systematic review of empirical tests. Soc Sci Med. 2022;292:114523. 34785096 10.1016/j.socscimed.2021.114523

[12] Lumivero. inventor NVivo (version 12 pro)2017.

[13] Braun V, Clarke V. Using thematic analysis in psychology. Qualitative Res Psychol. 2006;3(2):77–101.

[14] Gale NK, Heath G, Cameron E, Rashid S, Redwood S. Using the framework method for the analysis of qualitative data in multi-disciplinary health research. BMC Med Res Methodol. 2013;13(1):117. 24047204 10.1186/1471-2288-13-117 PMC3848812

[15] Burden M, Mooney PD, Blanshard RJ, White WL, Cambray-Deakin DR, Sanders DS. Cost and availability of gluten-free food in the UK: in store and online. Postgrad Med J. 2015;91(1081):622–6. 26310267 10.1136/postgradmedj-2015-133395

[16] Singh J, Whelan K. Limited availability and higher cost of gluten-free foods. J Hum Nutr Dietetics. 2011;24(5):479–86. 10.1111/j.1365-277X.2011.01160.x 21605198

[17] Sugavanam T, Crocker H, Violato M, Peters M. The financial impact on people with coeliac disease of withdrawing gluten-free food from prescriptions in England: findings from a cross-sectional survey. BMC Health Serv Res. 2024;24. 10.1186/s12913-024-10600-4 PMC10826048 38287389

[18] Muhammad H, Reeves S, Ishaq S, Mayberry J, Jeanes Y. Adherence to a gluten free diet is associated with receiving gluten free foods on prescription and Understanding food labelling. Nutrients. 2017;9(7):705. 28684693 10.3390/nu9070705 PMC5537820

[19] Peters M, Crocker H, Jenkinson C, Violato M. Withdrawing gluten-free food from prescriptions in england: a mixed‐methods study to examine the impact of policy changes on quality of life. J Hum Nutr Dietetics. 2020;33(4):453–64. 10.1111/jhn.12728 PMC7383817 31876360

[20] NHS England. Prescribing gluten-free foods in primary care: guidance for clinical commissioning groups 2018 [Available from: https://www.england.nhs.uk/wp-content/uploads/2018/11/prescribing-gluten-free-foods-primary-care-guidance-for-ccgs.pdf

[21] Croker R, Walker AJ, Bacon S, Curtis HJ, French L, Goldacre B. New mechanism to identify cost savings in english NHS prescribing: minimising ‘price per unit’, a cross-sectional study. BMJ Open. 2018;8(2):e019643. 29439078 10.1136/bmjopen-2017-019643 PMC5829890

[22] Saturni L, Ferretti G, Bacchetti T. The gluten-free diet: safety and nutritional quality. Nutrients. 2010;2(1):16–34. 22253989 10.3390/nu20100016 PMC3257612

[23] Lerner A, O’Bryan T, Matthias T. Navigating the gluten-free boom: the dark side of gluten free diet. Front Pead. 2019;7. 10.3389/fped.2019.00414 PMC6803387 31681712

[24] Melini V, Melini F. Gluten-Free diet: gaps and needs for a healthier diet. Nutrients. 2019;11(1):170. 30650530 10.3390/nu11010170 PMC6357014

[25] Trott N, Holland W, Hoffmann O, Shiha MG, Raju SA, Penny HA, et al. Food related quality of life and associations with demographic and clinical characteristics in people with coeliac disease. J Hum Nutr Dietetics. 2025;38(3):e70051. 10.1111/jhn.70051 40289367

[26] Pinto-Sanchez MI, Verdu EF, Gordillo MC, Bai JC, Birch S, Moayyedi P, et al. Tax-Deductible provisions for Gluten-Free diet in Canada compared with systems for Gluten-Free diet coverage available in various countries. Can J Gastroenterol Hepatol. 2015;29(2):104–10. 25803021 10.1155/2015/508156 PMC4373559

[27] Allen B, Orfila C. The availability and nutritional adequacy of Gluten-Free bread and pasta. Nutrients. 2018;10(10):1370. 30257431 10.3390/nu10101370 PMC6213709

[28] Jeanes Y, Rasheid R, Hovland C, Costabile A. Nutrient composition of commercially available compared with prescription only gluten-free bread and flour mixes in England. Proc Nutr Soc. 2020;79(OCE2):E437.

[29] Coeliac UK. New Coeliac UK report sheds light on cost, access & availability of gf food 2024. Available from: https://www.coeliac.org.uk/about-us/media-centre/news/new-coeliac-uk-report-sheds-light-on-cost-access-and/

[30] Coeliac UK. Prescriptions 2024. Available from: https://www.coeliac.org.uk/information-and-support/coeliac-disease/once-diagnosed/prescriptions/

[31] Shani M, Van Zalk MHW. Love beyond gluten: self-esteem, illness identity, and social support in romantic rejection concerns among adolescents with Celiac disease. Front Psychol. 2024;15. 10.3389/fpsyg.2024.1335201 PMC11144883 38831945

