# Supplementary material for: Patients’ views on a subsidy card model for gluten-free food access: a qualitative study
Source: BMC Health Serv Res. 2025 Oct 29;25:1418. doi: 10.1186/s12913-025-13582-z (PMC12570531; doi:10.1186/s12913-025-13582-z)
Supplement: Supplementary file 1 — Supplementary Material 1 [file 12913_2025_13582_MOESM1_ESM.docx]

# Supplementary Material 1-COREQ (COnsolidated criteria for REporting Qualitative research) Checklist

| **Domain/Item** | **Item Description** | **Reported on Page** | **Notes** |
| --- | --- | --- | --- |
| **Domain 1: Research team and reflexivity** - Interviewers | Which author(s) conducted the interview? | Methods | Interviews were conducted by Abubakar Sha’aban and Francesca Mazzaschi |
| Credentials | What were the interviewer’s credentials? | Methods | PhD |
| Occupation | What was their occupation at the time of the study? | Methods | Research Associates |
| Gender | Was the researcher male or female? | Methods | AS (Male), FM (Female) |
| Experience and training | What experience or training did the researcher have? | Methods | Formal training and experience in qualitative interviewing |
| Relationship established | Was a relationship established prior to study commencement? | Methods | No |
| Participant knowledge of the interviewer | What did participants know about the researcher? | Methods | Informed that interviewers were researchers studying healthcare access and patient perspectives |
| Interviewer characteristics | What characteristics were reported about the interviewer? | Methods | Trained researchers interested in service improvement in Health and Care Research Wales |
| **Domain 2: Study design -** Methodological orientation | What methodological orientation was stated to underpin the study? | Methods | Qualitative approach; thematic analysis (Braun & Clarke, 2006) |
| Sampling | How were participants selected? | Methods | Purposive sampling |
| Method of approach | How were participants approached? | Methods | Recruitment poster via social media (X, Facebook) and Coeliac UK mailing list |
| Sample size | How many participants were in the study? | Results | 23 participants |
| Non-participation | How many people refused or dropped out? | Methods | Of the 40 participants approached, 17 did not progress to the interview stage due to non-return of consent forms or because recruitment had reached data saturation. |
| Setting of data collection | Where was the data collected? | Methods | Online (Microsoft Teams/Zoom) |
| Presence of non-participants | Was anyone else present besides participants and researchers? | Methods | No |
| Description of sample | What are the characteristics of the sample? | Results | Gender, age range, disability, education, employment, prescription use status, income |
| Interview guide | Were questions/prompt guides provided? | Methods | Yes, developed and reviewed by public partner and expert |
| Repeat interviews | Were repeat interviews carried out? | Methods | No |
| Audio/visual recording | Did the research use recording? | Methods | Yes, with consent |
| Field notes | Were field notes made during and/or after the interview? | Methods | Yes, notes on peculiar situations after interviews |
| Duration | What was the duration of the interviews? | Methods | 30–45 minutes |
| Data saturation | Was data saturation discussed? | Methods | Yes, recruitment until no new themes |
| Transcripts returned | Were transcripts returned to participants? | Methods | No |
| **Domain 3: Analysis and findings -** Number of data coders | How many data coders coded the data? | Methods | Two (AS and FM) |
| Description of the coding tree | Did authors provide a description of the coding tree? | Methods | Yes, framework developed iteratively |
| Derivation of themes | Were themes identified in advance or derived from data? | Methods | Inductively derived |
| Software | What software was used to manage the data? | Methods | NVivo 12 |
| Participant checking | Did participants provide feedback on findings? | Methods | No |
| Quotations presented | Were participant quotations presented to illustrate themes? | Results | Yes |
| Data and findings consistent | Was there consistency between data presented and findings? | Results | Yes |
| Clarity of major themes | Were major themes clearly presented? | Results | Yes |
| Clarity of minor themes | Is there a description of diverse cases/minor themes? | Results | Yes |
