# Supplementary material for: Patients’ views on a subsidy card model for gluten-free food access: a qualitative study
Source: BMC Health Serv Res. 2025 Oct 29;25:1418. doi: 10.1186/s12913-025-13582-z (PMC12570531; doi:10.1186/s12913-025-13582-z)
Supplement: Supplementary file 2 — Supplementary Material 2 [file 12913_2025_13582_MOESM2_ESM.docx]

**Supplementary Material 2: Interview Guide**

**Topic Guide** (please note that the exact questions will be iterated depending on the discussion with the participant and throughout ongoing thematic analysis)

**Research Question 1: *How satisfied are you with the services you currently receive?***

1. Do you feel that you currently have access to high quality gluten-free food?
2. Do you feel that you currently have access to enough gluten-free food
3. Are you happy with the variety of gluten-free foods available (or received)?
4. Are you happy with the care provided by your health care team regarding your coeliac disease?

**Research Question 2: *How does your current method of accessing gluten-free food impact your daily life?***

1. How easy is it for you to access the gluten-free food you need?
2. Does accessing gluten-free food require additional travel?
3. Do you feel that your financial situation would be different if you did not require gluten-free food?
4. Is accessing gluten-free food easily incorporated into your daily routine?

**Research Question 3: *What are the perceived benefits or disadvantages of switching to using a pre-paid credit card?***

1. What would you say is the most beneficial aspect of using prescriptions?
2. What would you say is the biggest disadvantage of using prescriptions?
3. Is there anything you would like to change about the way you currently access gluten free products?
4. What do you think would be the main advantages and disadvantages of using a subsidy card to access gluten free products?
5. If you were offered the subsidy card scheme, would you want to switch?

**Research Question 4: What are your main priorities regarding access to gluten free products?**

1. What is most important to you in terms of accessing gluten free products?
2. Do you think anything could be done to improve how you access gluten free products?
